# Simulation of the Mechanical Behaviour of Metal Gyroids for Bone Tissue Application

**DOI:** 10.3390/ma14174808

**Published:** 2021-08-25

**Authors:** Fabrizia Caiazzo, Diego Gonzalo Guillen, Vittorio Alfieri

**Affiliations:** Department of Industrial Engineering, University of Salerno, Via Giovanni Paolo II 132, 84084 Fisciano, Italy; f.caiazzo@unisa.it (F.C.); dguillen@unisa.it (D.G.G.)

**Keywords:** gyroid, bone scaffolds, simulation, validation, FEM

## Abstract

Additive manufacturing is a valid solution to build complex geometries, including lightweight structures. Among these, gyroids offer a viable concept for bone tissue application, although many preliminary trials would be required to validate the design before actual implantation. In this frame, this study is aimed at presenting the background and the steps to build a numerical simulation to extract the mechanical behaviour of the structure, thus reducing the experimental effort. The results of the simulation are compared to the actual outcome resulting from quasi-static compressive tests and the effectiveness of the model is measured with reference to similar studies presented in the literature about other lightweight structures.

## 1. Introduction

In recent years, lightweight cellular structures have become very popular for many applications, including biomedical and aerospace ones, thanks to the significant advantages they offer, such as weight reduction, high strength, good impact response and excellent energy absorption capacity [1]. When comparing traditional lightweight structures and solid materials, the superiority of TPMS (triple periodical minimal surface) structures can be observed in terms of higher specific strength and stiffness combined with greater freedom in structural design. TPMS structures are often presented as an organized sequence of elementary cells providing continuous deformation; the overall architecture can be properly designed with the purpose of increasing the mechanical resistance under external loads [2]. In the family of TPMS, the gyroid cellular structure, or the Schoen-G, is a special geometry offering greater correspondence with a trabecular human bone, compared to other TPMSs, such as the diamond or Nevious surface [3]. Indeed, the specific porous configuration provides an optimal area for proliferation of totipotent cells, thus resulting in good biocompatibility [1,3]; given these reasons, the gyroid geometry is the most favourable to produce scaffolds for human implants, compared to other TPMSs. Nevertheless, due to the geometric complexity of the cells, the fabrication process of a gyroid scaffold is challenging with conventional technologies. Additive manufacturing, instead, and the technology of laser powder bed fusion (LPBF) of metals in particular have shown significant potential for the fabrication of complex structures, including TPMSs [4], thanks to making efficient use of raw materials and producing minimal scrap while achieving satisfactory geometrical accuracy [3,4]. Interestingly, gyroids cells are self-supporting, thus preventing the need for specific supporting structures [5], which should be removed after building. In addition, customer-oriented solutions can be pursued for missing or injured bones. Different studies have already been published, aiming at discussing the surface morphology, the mechanical properties and the manufacturability of metal gyroids via additive manufacturing [6]. It has been pointed out that many geometrical factors are involved and affect the response under loading [5]. In this frame, the results of the quasistatic compressive behaviour as a function of wall thickness and orientation of the main cells have been widely discussed [2,6] for biomorphic gyroid scaffolds of different metal alloys. More specifically, it has been shown that the anisotropy of the gyroid structure can be conveniently designed to reduce stress shielding after surgery [6]; indeed, functionally graded gyroids structures have been obtained by means of linearly shifting the pore size and the surface area of the elementary cell in the direction of loading, for a given constant porosity [7]. Moreover, matrix phase gyroids and network phase gyroids have been also proposed as variations of the base scheme, providing the desired volume fraction with a simple linear equation [8]. At this point, a wide experimental campaign would be required to effectively take account of each geometrical factor; moreover, since a customer-oriented application is aimed at, an effective and reliable method to predict the mechanical behaviour of the gyroids would provide a crucial tool to reduce time and cost of the preliminary trials. Numerical simulations are valuable solutions, since different scenarios can be implemented by setting proper decision rules and operating variables; then, the process of finding the optimum design solution can be virtually performed in accelerated time, compared to the actual complete trial. In this sense, the finite elements method (FEM) is among the most effective and powerful numerical techniques for solving partial differential problems in different areas of mathematics, physics and engineering, in general. Indeed, the approach has been successfully used to study lightweight structures in general [9,10]. An elasto-viscoplastic model has been built [11] to compare the gyroid structure to other TPMS, concluding that better capacity of minimization of stress concentration is offered by the gyroids. It is worth noting that different degrees of precision of the simulations have been achieved in the literature regarding the mechanical behaviour of lightweight structures; a short survey of these is useful to set a benchmark for further assessment of other simulations, including the model proposed here. For a polycarbonate sandwich structure with a corrugated bi-directional core made by fused filament fabrication, then bonded with epoxy adhesive at controlled temperature [12], a mismatch in the order of 14% has been obtained for the elastic modulus with respect to the analytical solution, which was affected by an additional 6% error with respect to the experimental result. The nodal displacement method has been proposed [13] to study three types of self-supporting AlSi10 Mg lattice structures made by LPBF, with 0.6 mm wall thickness; in this case, the mismatch between the FEM-predicted and the experimental elastic modulus was found in a range between −7.2 and 8%. The FEM approach has been also considered to compare the mechanical properties of as-built and heat-treated stainless steel parts made via directed-energy deposition, or LPBF [14]; namely, the model provided an error of up to 38% on the elastic modulus in the heat-treated state. It has been reported that the study fails in accounting for the manufacturing anisotropies, which are typical of the fabrication technique. Given this background, the target of this work is to build up an FEM-based numerical model to simulate the mechanical behaviour of different gyroid structures and validate the outcome based on experimental trials. The aim is presenting the hypothesis, the approach for meshing, the conditions and any additional useful information to guide a researcher in performing a simulation of a representative gyroid structure for each potential application.

## 2. Experimental Procedures

### 2.1. Design of the Samples

**Figure 1:**
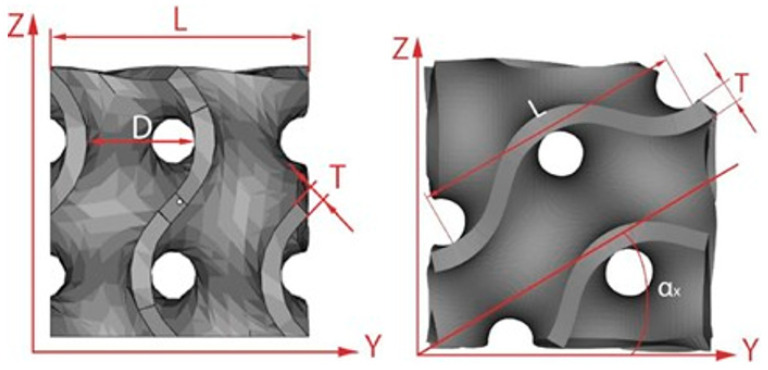
Geometrical factors of an elementary unit cell.

Many geometrical factors (Figure 1) are involved in the description of a gyroid cellular structure [9], which is expressed by the equations

*G* = [*cos*(*k_x_x*)*sin*(*k_y_y*) + *cos*(*k_y_y*)*sin*(*k_z_z*) + *cos*(*k_z_z*)*sin*(*k_x_x*)]^2^ − *t*^2^ (1)

*k_i_* = 2*n_i_*/*L_i_*; *i* = *x*, *y*, *z*

Here, the following symbols are used: *x*, *y* and *z* are the space directions, *n_i_* the number of repetitions of the wave and *k_i_* the scale factor along each coordinate axis. To set the thickness t to manufacture the surface, which consequently determines the volume fraction of the resulting structure, two different methods are possible [8], creating a mesh phase by joining the unconnected faces, or giving a normal thickness to each face of the surface; the latter was used here. Based on the independent geometrical factors, the void size can be found as follows:

*d_i_* = *L_i_*/2 − *t* (2)

Eventually, the orientation sets the direction of arrangement of the elementary units with respect to each axis, thus affecting the void size and resulting in structural anisotropies.

In this study, a constant wavelength of 7.5 mm was chosen; two values of wall thickness (0.4 and 0.6 mm, in agreement with the resolution of the actual fabrication process) and three orientations (0, 40 and 45°) were selected; the scale factor was applied only along the z axis. Six conditions (Table 1) were generated, in a mixed experimental plan; the driving idea was to test the validity of the simulation tool over different schemes, aiming at exploring its effectiveness in dealing with three crucial geometrical factors affecting the global structure and the response in turn.

### 2.2. Virtual SAMPLES

The commercial software nTop Platform (3.0, nTopology, New York, NY, USA), which is specifically conceived for creating TPMS structures with reduced file size and optimized algorithms, was used to generate the virtual specimens in the selected conditions of the plan. More specifically, 15 mm size cubes (subsize specimens) were designed by replicating the elementary units for each condition; 30 mm size cubes, the same as the actual samples, were additionally designed for conditions 1, 4 and 5 to assess the impact in terms of time for solving and reliability of the simulation. Surface meshing was accomplished by nTop as well, using triangular elements with an average size of 0.5 mm; this first step aimed at generating a first-stage discretization of the continuous surface of each virtual sample. A second step of remeshing was required afterwards, namely, the size of each element was subsequently reduced to 0.2 mm for the purpose of increasing the spatial resolution in preparation for the step of volumetric meshing. Eventually, the mesh was exported in .3mf format to feed the software for numerical analysis.

### 2.3. Numerical Solution

The commercial software Comsol Multiphysics (5.3, COMSOL Inc., Burlington, MA, USA) was selected as numerical solver, thanks to its potential and flexibility. Many steps were required to build the model and are addressed in the following.

#### 2.3.1. Material and Physics Background

**Figure 2:**
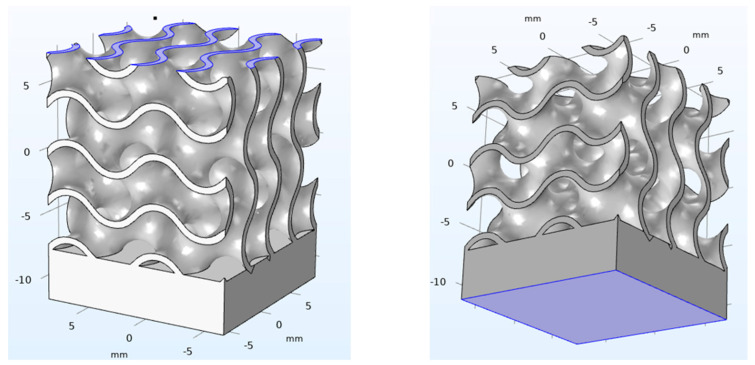
Boundary conditions applied at the upper and lower surface of each virtual specimen.

The required material library was not native of the solver; therefore, the basic information regarding the mechanical properties (i.e., Young’s modulus, yield strength, Poisson ratio and tangential isotropic module) were collected in the literature [15,16] and fed to the software. Structural mechanics was selected as the main physic of the problem. Since the study is intended for applications where reversible deformations apply, a model of linear elasticity was implemented; nevertheless, to provide a complete description of the loading phase, the non-linear stage was modelled using the elastoplastic theory, where the constitutive relations of the von Mises theory were considered. More specifically, the hypothesis of linear isotropic hardening was implemented. Regarding the boundary conditions (Figure 2), prescribed displacements in the quasi-static compression tests were given via a Dirichlet condition [17], namely, a fixed constraint of zero displacement was set to the upper face of the specimen, whereas a pre-set speed of 1 mm/min was set to the opposite face, thus simulating the external compressive load under a condition of displacement control, according to previous studies [5,18]. Two virtual probes, for the displacement and the instant stress at the upper surface, were defined and monitored. To detect the response of each probe, the analysis was performed with a time step of 0.1 s over a monitoring time of 60 s.

#### 2.3.2. Creation of the Volumetric Mesh

Since the surface mesh created at the previous stage using nTop only represented a 2D mesh over a 3D volume of a curved surface, an upgrade to a volumetric mesh was required to solve the problem. This step was accomplished in Comsol and a free tetrahedral mesh, specific for the main physic of the problem, was chosen (Table 2). Namely, the geometric order of the mesh elements was second (quadratic elements); indeed, due to the geometric complexity of the structure, the quadratic shape function for discretization offered a better representation, thus leading to increased accuracy. The procedure was implemented for each virtual specimen.

**Figure 3:**
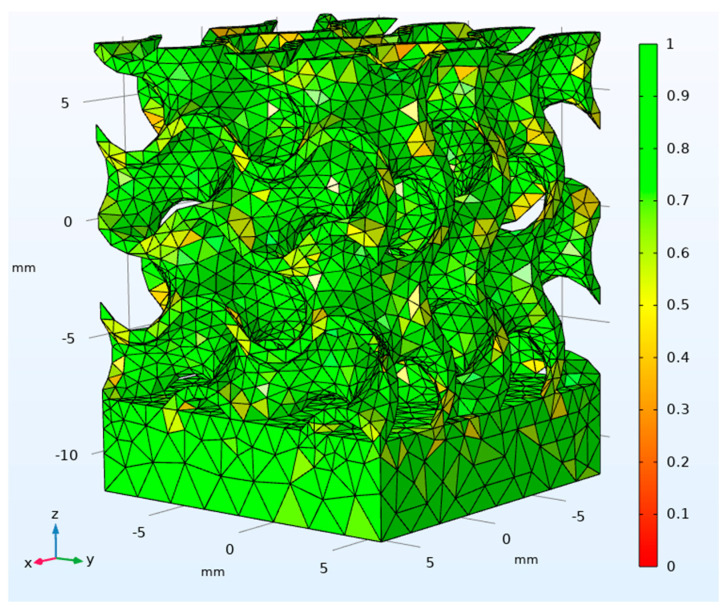
Skewness of the volumetric mesh for the 15 mm size cube in condition 1.

The quality of the mesh was assessed using the skewness of the discretization, which is the default measure for many mesh types, ranging from 0 (in case of a degenerated element) to 1 (in case of an ideal one); this was based on angular equi-symmetry, therefore elements with large or small angles with respect to the angles of an ideal element were penalized (Figure 3). Based on this, an average value of skewness could be given to each virtual specimen; the total amount of elements of each mesh depended on the extent of the specimen; the size of the file and the time for solving were affected in turn (Table 3).

#### 2.3.3. Actual Samples

The same conditions selected for the virtual specimens were considered for manufacturing the actual samples, which are part of a wider experimental plan conducted by the authors to investigate the effect of the geometrical factors on the mechanical properties of biomorphic scaffolds [5]. Namely, the actual samples were 30 mm size cubes and were manufactured via LPBF in full melting mode (Table 4) using a commercial machine EOSINT M270 (EOS GmbH, Krailling, Germany) with pre-alloyed stainless- steel UNS S17400 powder by the same manufacturer; this chemical was chosen since it offers biocompatibility for bone implants, as widely discussed in the literature [3,6].

The response variables selected to assess the reliability of the simulation were measured in compressive quasi-static tests using an MTS Landmark servo-hydraulic machine (MTS, Eden Prairie, MN, USA), at a sampling rate of 3 Hz, in condition of displacement control at 1 mm/min speed, up to 6% deformation.

## 3. Results

**Figure 4:**
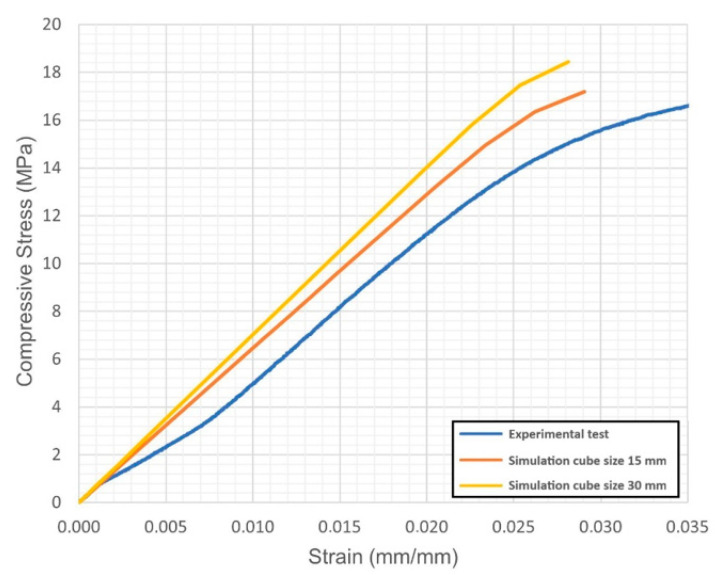
Condition 1, stress-strain diagram; comparisons between simulated and actual specimens.

**Figure 5:**
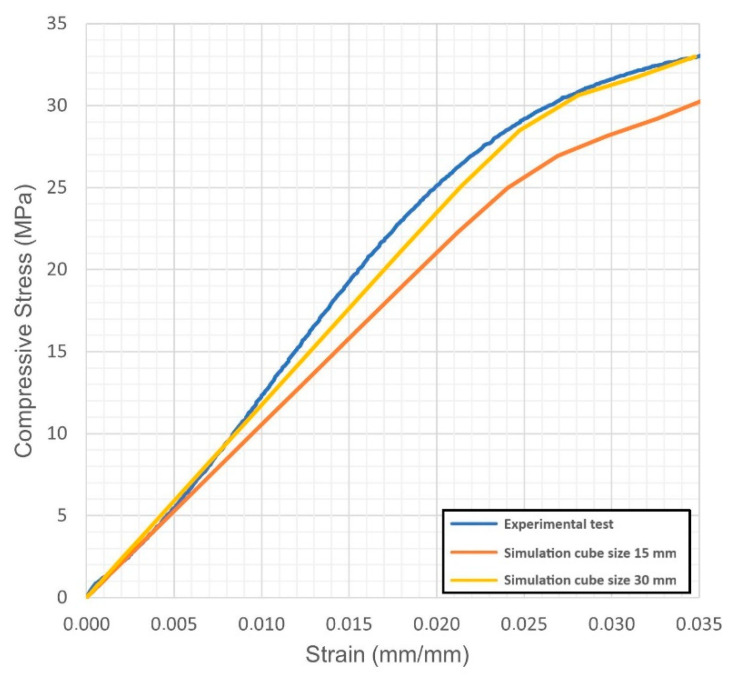
Condition 4, stress-strain diagram; comparisons between simulated and actual specimens.

**Figure 6:**
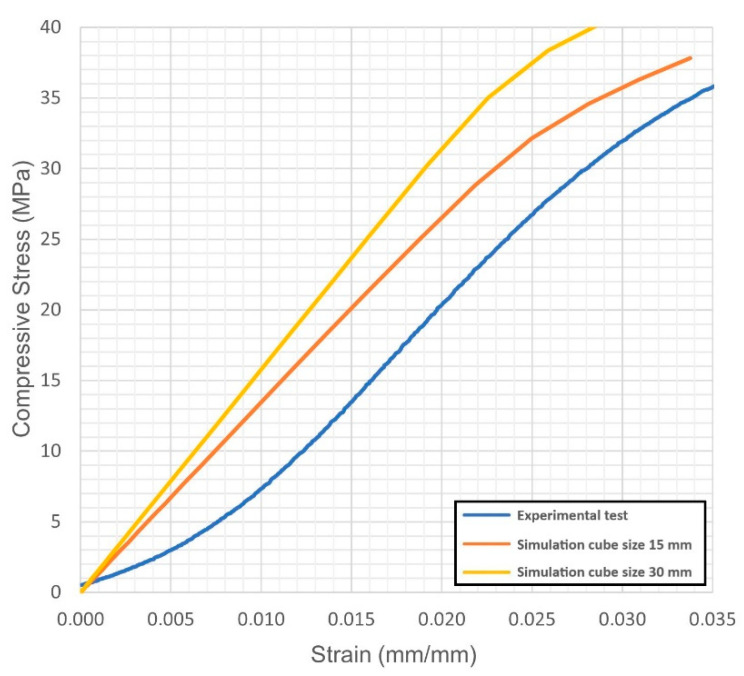
Condition 5, stress-strain diagram; comparisons between simulated and actual specimens.

**Figure 7:**
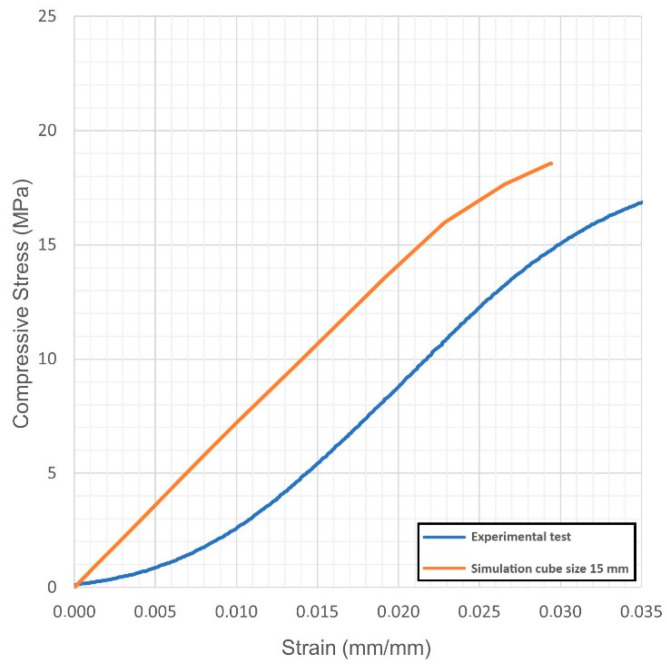
Condition 2, stress-strain diagram; comparisons between simulated and actual specimens.

**Figure 8:**
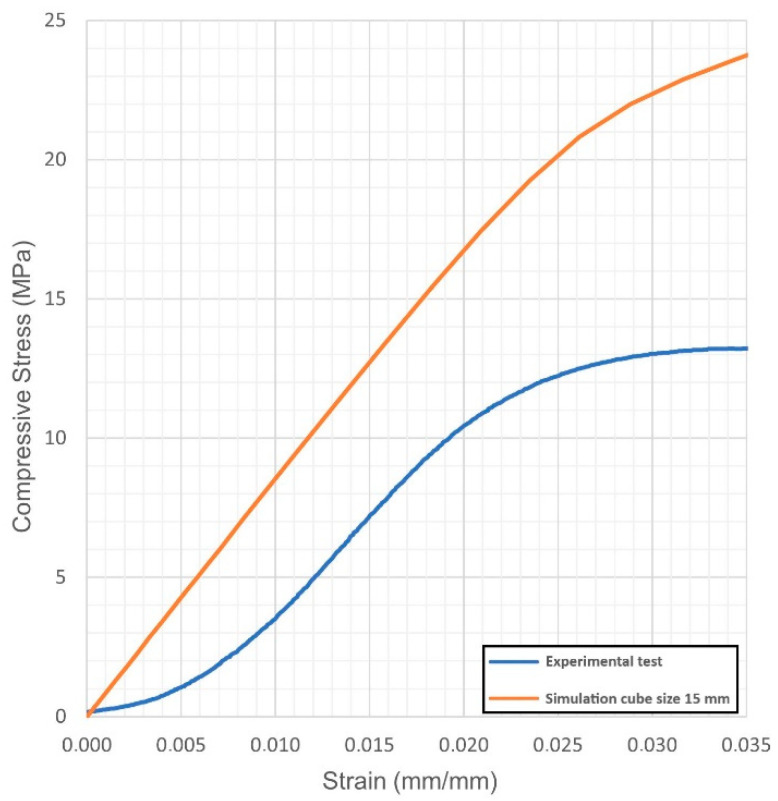
Condition 3, stress-strain diagram; comparisons between simulated and actual specimens.

**Figure 9:**
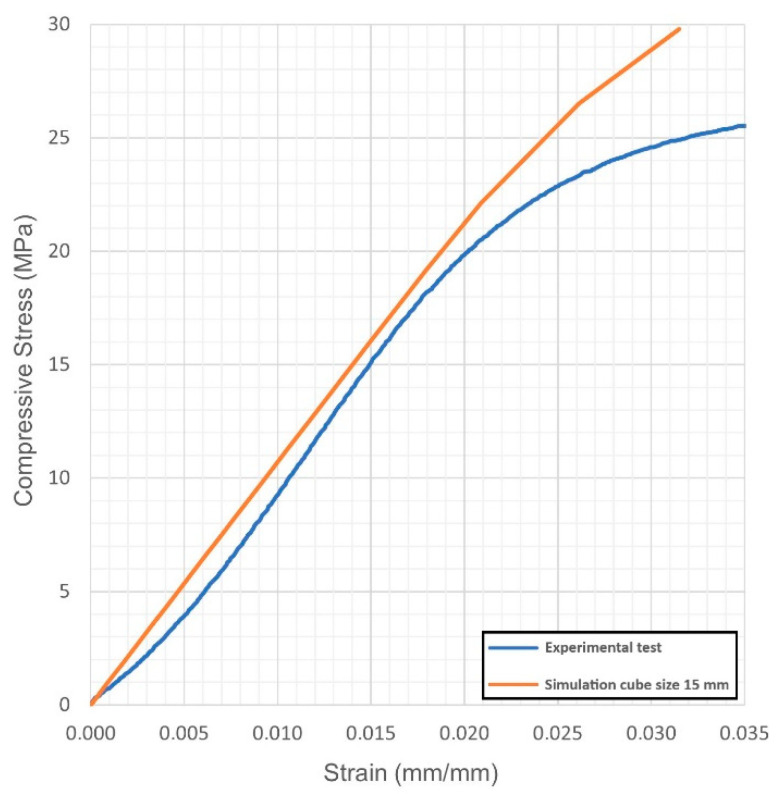
Condition 6, stress-strain diagram; comparisons between simulated and actual specimens.

For each virtual sample, the stress-strain diagrams were obtained; based on these, the Young’s modulus and the yield strength were evaluated. The outcome was compared with the actual values resulting from quasi-static compression tests (Figure 4, Figure 5, Figure 6, Figure 7, Figure 8 and Figure 9, Table 5). Regarding the stress-strain diagrams, it is worth noting that, as reported in the literature [18], test initiation must allow the plate of the cross-head of the testing machine to reach a condition of full contact with the surface of the sample, before the linear elastic stage is found; clearly, this is not required in the simulation where ideal conditions apply. Due to this, the diagram of the actual behaviour is right-shifted with respect to the simulated outcome. However, both in the simulated and the experimental trend, the yield strength was evaluated at a 0.2% strain offset with respect to the elastic stage.

At first, the impact of the size of the virtual specimen was discussed; therefore, conditions 1, 4 and 5 were considered. For each geometry, the stress strain diagram and the mechanical properties in turn are affected when comparing the subsize virtual specimen to the actual size specimen. The reason is ascribed to the mesh quality; therefore, the mismatch is generally increased when the discretized volume is increased. Nevertheless, the average deviation in terms of mechanical properties was below 10% between the two approaches and below 9% with respect to the experimental values, whereas an average reduction of the 93% in terms of time for solving was benefited in simulating the subsize specimen. Based on this, it can be inferred that a subsize simulation is effective; indeed, a 15 mm size cube offered a representative sample when a 7.5 mm wavelength was designed. Average mismatches of 10% and 14% resulted for the Young’s modulus and the yield strength, respectively. These are generally lower than the deviations resulting from similar studies about simulation of the mechanical behaviour of other lightweight structures, as referred in the introduction. Possible reasons for the mismatch to the actual values can be supposed. At first, the material properties of the solver library were set as isotropic, whereas many studies reported anisotropy of LPBF-made parts as a function of the direction of building [19,20] and post-process heat treatments [21]; to implement a more accurate library, further trials on raw material and specific samples would be required. Moreover, it has been reported [5] that although a suitable accuracy of printing in the order of 99.5% is achieved in terms of wall thickness, local imperfections of layer adhesion may result in cell collapse, thus reducing the overall real strength, compared to the predicted mechanical resistance which was evaluated for the intended ideal structure.

## 4. Conclusions

The FEM approach developed in this study provided a reliable outcome which was validated based on experimental trials. Namely, the following findings are highlighted:

- the implicit design method used through nTop allowed to easily address the geometrical features of each sample and was effective in reducing common file errors due to the discretization of complex geometries;
- subsize virtual specimens can be effectively considered, provided that a representative number of cells is set; in this study, a 15 mm size cube was adequate, since a nominal wavelength of 7.5 mm was set;
- a reduction of the virtual volume to 1/8 (i.e., moving from a 30 mm to a 15 mm size cube) was effective in reducing the overall time for solving in a measure of more than 90%;
- the mismatch between actual and simulated values of mechanical properties was lower compared to similar studies on simulations of other lightweight structures in the literature;
- the deviations are ascribed to incomplete data libraries or local imperfections of the actual samples due to typical LPBF limitations.

Based on this, grounds are given for the numerical simulation of gyroids structures for case-by-case engineering and biomedical applications, where tissues must be properly designed to match the mechanical properties of the specific injured bone, depending on age, sex and global health conditions.

## Figures and Tables

**Table 1:** Geometrical factors for each condition.

Condition	Wall Thickness (mm)	Orientation (°)	Z-Axis Scale Factor	Void Frac- tion (%)	Elementary Unit
1	0.4	0	1	83.75	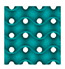
2	0.4	40	1	83.75	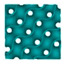
3	0.4	40	2	85.95	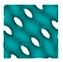
4	0.6	0	1	75.80	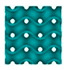
5	0.6	45	1	75.80	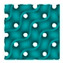
6	0.6	45	2	79.00	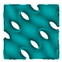

**Table 2:** Parameters of the tetrahedral mesh generated in Comsol.

Item	Value
Maximum element size	19.5 mm
Minimum element size	0.35 mm
Maximum growth rate of one element	1.5
Curvature factor	0.6
Resolution in narrow regions	0.5

**Table 3:** Surface and volumetric mesh description, with resulting quality, file size and time for solving.

Cond.	Cube Size (mm)	Triangular Elements	Tetrahedral Elements	Minimum Quality	Average Quality	File Size (GB)	Time for Solving (min)
1	15	34,168	22,373	0.04	0.66	12	10
	30	281,608	188,184	0.14	0.63	117	148
2	15	37,432	26,859	0.02	0.62	9	7
3	15	31,008	29,985	0.01	0.66	13	8
4	15	35,608	28,123	0.01	0.66	22	12
	30	295,512	182,039	0.07	0.64	189	155
5	15	39,696	29,102	0.07	0.63	10	11
	30	281,504	166,315	0.08	0.66	121	148
6	15	32,248	23,724	0.14	0.65	9	5

**Table 4:** Levels of the processing parameters in LPBF in full melting mode.

Factor	Value
Laser power (W)	195
Scanning velocity (m/s)	0.75
Scan length (mm)	20
Hatch spacing (µm)	100
Layer thickness (µm)	20

**Table 5:** Mechanical properties for each condition and percentage mismatch to the experimental values.

Cond.	Real Specimen	Virtual Specimen	Size of Virtual Specimen (mm)	Simulated Young’s Modulus (MPa)	Actual Young’s Modulus (MPa)	Mismatch on Young’s Modulus (%)	Simulated Yield Strength (MPa)	Actual Yield Strength (MPa)	Mismatch on Yield Strength (%)
1	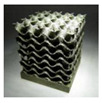	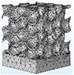	15	643	602	6.8	16.8	15.4	9.1
		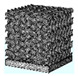	30	701	602	16.4	18.5	15.4	20.1
2	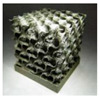	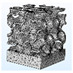	15	711	736	3.4	17.8	15.8	12.7
3	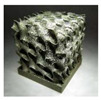	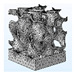	15	853	825	3.4	20.5	11.4	79.8
4	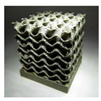	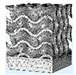	15	1179	1346	12.4	30.4	28.5	6.7
		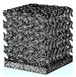	30	1203	1346	10.6	31.4	28.5	10.2
5	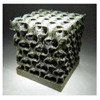	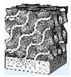	15	1347	1451	7.2	33.6	32.1	4.7
		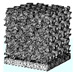	30	1540	1451	6.1	37.9	32.1	18.1
6	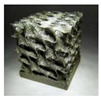	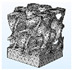	15	1070	1029	4.0	27.5	24.6	11.8

## Data Availability

All the data required to replicate the results are included in the paper; additional information is available on request from the corresponding author.
